# Dissemination of Carbapenemases and MCR-1 Producing Gram-Negative Bacteria in Aquatic Environments in Batna, Algeria

**DOI:** 10.3390/antibiotics11101314

**Published:** 2022-09-27

**Authors:** Zineb Cherak, Lotfi Loucif, Esma Bendjama, Abdelhamid Moussi, Amel Benbouza, Nadia Grainat, Jean-Marc Rolain

**Affiliations:** 1 Faculté des Sciences de la Nature et de la Vie, Université de Batna 2, Batna 05078, Algeria; 2 Laboratoire de Biotechnologie des Molécules Bioactives et de la Physiopathologie Cellulaire (LBMBPC), Faculté des Sciences de la Nature et de la Vie, Université de Batna 2, Batna 05078, Algeria; 3 Départements de Technologie Alimentaire, Instituts des Sciences Agronomiques et Vétérinaires, Université El Hadj Lakhdar-Batna 1, Batna 05000, Algeria; 4 Laboratoire de Génétique, Biotechnologie et Valorisation des Bioressources (GBVB), Faculté des Sciences Exactes et des Sciences de la Nature et de la Vie, Université Mohamed Khider, Biskra 07000, Algeria; 5 Faculté de Médecine, Université de Batna 2, Batna 05078, Algeria; 6 Faculté de Médecine et de Pharmacie, Aix Marseille Université, IRD, MEPHI, 13007 Marseille, France; 7 IHU Méditerranée Infection, 13005 Marseille, France; 8 Assistance Publique des Hôpitaux de Marseille, 13005 Marseille, France

**Keywords:** GNB, carbapenemases, *mcr-1*, water environments, Algeria

## Abstract

Antibiotic-resistant-bacteria are being considered as emerging environmental contaminants where the importance of the surrounding environment in their emergence and dissemination has been emphasized. The aim of this study was to screen for the presence and diversity of carbapenem- and colistin-resistant Gram-negative bacteria (GNBs) in different aquatic environments. Water samples were collected in Batna, Algeria. Carbapenem- and colistin-resistant GNBs were selectively isolated and then identified using matrix-assisted laser desorption and ionization time-of-flight mass spectrometry. After phenotypic antibiotic susceptibility testing, the molecular mechanisms of β-lactams and colistin-resistance were investigated by PCR and sequencing. The clonality of *mcr-1* positive *Escherichia coli* was determined by multi-locus sequence typing. We noticed a high level of resistance in both tap water and wastewater. The most commonly found carbapenem-resistance mechanism was the OXA-48 enzyme, but other carbapenemases were also detected. In addition, the *mcr-1* gene was detected in 18 *E. coli* of different sequence types. Our findings highlight the role of aquatic environments in the dissemination of resistant-bacteria, especially considering that water is a connecting medium between different ecological systems and can easily transmit resistant-bacteria and promote horizontal gene transfer. Thus, the development of effective treatment strategies for eliminating antibiotic-resistance is seriously needed.

## 1. Introduction

The discovery of antibiotics and their use for the treatment of bacterial infections marked one of the greatest breakthroughs in human history [1]. However, bacterial resistance to these compounds has rapidly emerged and now represents a major concern to public health, social progress, and food security [2]. This phenomenon is associated with increased mortality and costs, where the World Health Organization (WHO) estimates that by 2050, ten million deaths and approximately US$100 trillion will be spent every year as a result of antimicrobial resistance [1]. Although commonly considered as a healthcare-associated problem, community acquired infections due to antibiotic resistant bacteria are increasing, and reports of bacterial resistance even to last resort antibiotics are on the rise [3]. In recent years the importance of the surrounding environment in the emergence and dissemination of drug-resistant bacteria has been emphasized, especially within the concept of the “One Health” approach, which was launched in September 2017 https://www.who.int/news-room/questions-and-answers/item/one-health (accessed on 15 August 2022). On the same note, the WHO highlighted the need for further investigations regarding the routes of antibiotic resistance transmission, including water and the natural environment, in its report *Global Action Plan on Antimicrobial Resistance* [4].

Water is known to be one of the most important microbial habitats where bacteria from different sources may mix, allowing the circulation and exchange of genes including those encoding for antibiotic resistance mechanisms [5,6]. Aquatic environments are potential reservoirs for multidrug resistant bacteria, which is clearly revealed by the multiplicity of studies reporting the detection of multidrug resistant bacteria in water of different sources including drinking water, surface water, groundwater and wastewater. Each water type possesses some specifications allowing it to act as a reservoir and vehicle of adapted drug resistant bacteria [7]. Consequently, the investigation of drug resistant organisms in the different water types seems to be useful for understanding their epidemiology.

Carbapenems and colistin are bactericidal agents that are considered to be last line agents in the treatment of Gram-negative bacterial infections [8,9]. However, resistance to these compounds is increasingly reported and poses a serious public health concern. Carbapenem resistance in Gram-negative bacteria (GNB) is the result of an alteration to the outer membrane permeability or of enzymatic inactivation of the carbapenem through the production of hydrolytic enzymes known as carbapenemases, which are mainly encoded by mobile genetic elements [10]. Colistin resistance arises either from chromosomal mutations leading to lipopolysaccharide (LPS) modifications or loss, or from the expression of *mcr* (mobile colistin resistance) genes, resulting in modification of the bacterial LPS with phosphoethanolamine residues [11].

Carbapenem- and colistin-resistant GNB isolates are increasingly reported from different origins from diverse locations throughout Algeria, indicating their possible widespread dissemination across the country. An important aspect of combating antibiotic resistance is the assessment of aquatic environments as reservoirs of antibiotic-resistant bacteria and their roles in disseminating resistance to last resort antibiotics such as carbapenems and colistin. This study was carried out to screen for carbapenem- and colistin-resistant Gram-negative bacteria (GNB) in three types of domestic water including hospital tap water, hospital sewage, and wastewater released into the environment, and to investigate their molecular determinants.

## 2. Results

During the study period, a total of 445 GNB isolates were recovered, including 103 (23.15%) strains from wastewater samples (*n* = 35) and 342 (76.85%) from tap water and well water samples (*n* = 168 + 4) (Figure S1A,B). The results are summarized in File S1 (Excel file with four sheets).

### 2.1. Identification

#### 2.1.1. Tap Water and Well Water

Of the 342 recovered strains, we identified 126 (36.84%) *Enterobacterales* and 216 (63.16%) glucose-non fermenting GNB (Gnf-GNB) strains. With the exception of one *Enterobacterales* strain, all tap water and well water isolates were successfully identified by MALDI-TOF MS with a reliable identification score (>2). The remaining isolate could not be identified (identification score < 1.7) and was confirmed as new species throughout 16S rRNA sequencing. The identified enterobacteria belonged to six genera, namely *Escherichia* (7.93%), *Klebsiella* (37.3%), *Citrobacter* (5.55%), *Cronobacter* (0.79%), *Leclercia* (0.79%) and the most dominant genus, *Enterobacter* (47.82%). Glucose non-fermenting strains belonged to the following genera: *Cupriavidus* (0.46%), *Comamonas* (0.46%), *Stenotrophomonas* (5.09%), *Acinetobacter* (6.48%), *Aeromonas* (8.79%), and *Pseudomonas* not *P. aeruginosa* (23.61%), and the most dominant species was *P. aeruginosa* (55.09%) (Figure S1C).

#### 2.1.2. Wastewater

A total of 103 strains were isolated from wastewater samples on selective media, including 76 enterobacteria (73.78%) and 27 glucose non-fermenting GNBs (26.21%). *Enterobacterales* isolates belonged to nine genera, namely *Lelliottia*, *Raoultella*, *Kluyvera*, *Leclercia*, *Providencia*, *Enterobacter*, *Klebsiella*, *Citrobacter* and *Escherichia*. Regarding glucose-non fermenting GNBs, species belonging to the genera *Aeromonas*, *Acinetobacter*, *Comamonas*, *Shewanella* and *Pseudomonas* were identified (Figure S1A,D).

### 2.2. Antibiotic Susceptibility Testing

#### 2.2.1. Tap Water and Well Water

Despite the large number of isolates, a limited number of strains demonstrated antibiotic resistance phenotypes. The antibiogram results are presented in Figure S2A,B. Regarding enterobacteria and aeromonads, the highest resistance level was observed with cefalotin (59.31%) and amoxicillin-clavulanate (47.58%). Two strains were resistant to ertapenem (1.38%), including one, identified as *Citrobacter braakii*, which was imipenem resistant. None of the isolates was resistant or intermediately resistant to amikacin. The highest level of resistance among the Gnf-GNBs was noted to be against cotrimoxazole (88.83%), followed by ticarcillin (46.70%) and ticarcillin-clavulanate (30.45%). In addition, twenty-four strains were resistant to imipenem (12.18%), including a colistin resistant *Cupriavidus gilardii* isolate.

#### 2.2.2. Wastewater

Unlike tap water, wastewater isolates showed high antibacterial resistance levels, as they were all isolated on selective media. Antibiogram results are presented in Figure S2C,D. With regard to enterobacteria and aeromonads, 61 (74.39%) strains were resistant to amoxicillin-clavulanate, 59 (71.95%) to ertapenem, 57 (69.51%) to cotrimoxazole, 54 (65.85%) to cefotaxime and ciprofloxacin, 53 (64.63%) to cefoxitin and ceftazidime, 46 (56.09%) to cefepime, 41 (50%) to aztreonam, 39 (47.56%) to gentamicin, 28 (34.14%) to tobramycin, 23 (28.04%) to imipenem, 21 (25.60%) to colistin and, finally, seven were resistant to amikacin (8.53%). Among the Gnf-GNBs, 19 strains were resistant to cotrimoxazole, 18 to ticarcillin, 16 to imipenem, 15 to ticarcillin-clavulanate, 13 to piperacillin, 10 to ciprofloxacin, nine to piperacillin-tazobactam, ceftazidime, cefepime, tobramycin and gentamicin, and eight to aztreonam. Only one strain was resistant to amikacin, and no isolate was colistin-resistant.

### 2.3. Molecular Characterization of Antibiotic Resistance Mechanisms

#### 2.3.1. Tap Water and Well Water

**Figure 1:**
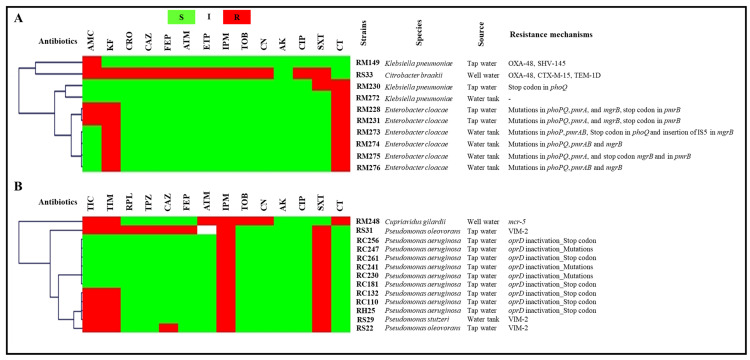
Antibiogram results and resistance mechanisms of tap water and well water isolates clustered using the MultiExperimentViewer (MEV) software version 4_6_2. (**A**), enterobacteria; (**B**), glucose-non-fermenting GNB. AK, amikacin; AMC, amoxicillin/ clavulanate; ATM, aztreonam; CAZ, ceftazidime; CIP, ciprofloxacin; CN, gentamicin; CTX, cefotaxime; CT, colistin; ETP, ertapenem; FEP, cefepime; I, intermediate; IPM, imipenem; KF, cefalotin; R, resistant; S, susceptible; SXT, cotrimoxazole; TOB, tobramycin; Strains’ codes: RC, cancer hospital; RH, university hospital; RM, maternity hospital; RS, public hospital.

Of the 342 obtained strains, carbapenem- and colistin-resistance mechanisms were detected in 23 strains. The identification, resistance profiles and detected resistance mechanisms of these latter are summarized in Figure 1. Carbapenem resistance was mostly related to *oprD* gene inactivation, which was detected in nine *Pseudomonas aeruginosa* isolates obtained from taps in the cancer hospital and the university hospital. Carbapenemase production was also noticed in two *Pseudomonas oleovorans* and one *Pseudomonas stutzeri* isolate recovered from the taps and water tank of the public hospital and were VIM-2 producers. OXA-48 carbapenemase was detected in two enterobacteria isolates, namely *Klebsiella pneumoniae* and *Citrobacter braakii* isolated from the maternity hospital tap water and the public hospital well, respectively.

Colistin resistance is often related to mutations in *phoPQ*, *pmrAB*, and *mgrB* genes. However, the mobile colistin resistance mechanism was also detected via the isolation of one *mcr-5*-producing *Cupriavidus gilardii* strain from the well water of the maternity hospital. The whole genome of this strain was sequenced and has already been published [12].

#### 2.3.2. Wastewater

**Figure 2:**
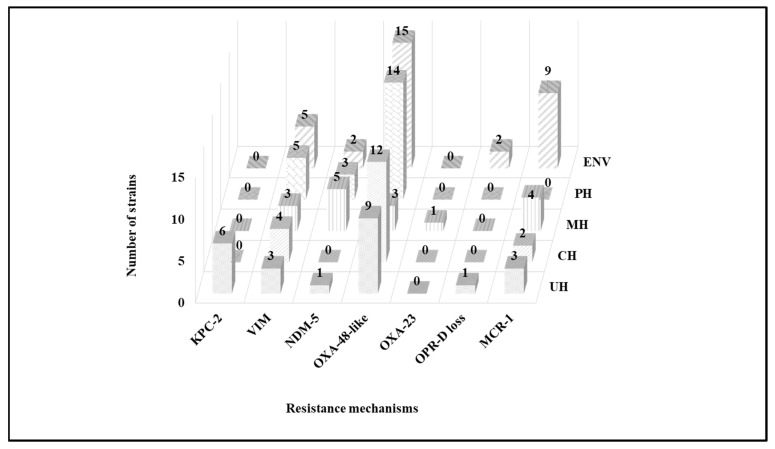
Detected β-lactam and colistin resistance mechanisms in wastewater by sampling site. CH, cancer hospital; ENV, environment; MH, maternity hospital; PH, public hospital; UH, university hospital.

The detected resistance mechanisms arranged by sampling site are presented in Figure 2. Carbapenemases of the three Ambler classes (A, B and D) were detected in the present study. Six strains identified as five *Aeromonas* spp. and one *Enterobacter cloacae* complex were KPC-2 producers. NDM-5 metallo-enzyme was detected in 11 enterobacterial isolates (10.68% of total wastewater isolates), namely six *Klebsiella pneumoniae*, two *Enterobacter cloacae* complex, one *Enterobacter asburiae*, one *Klebsiella oxytoca* and one *Leclercia adecarboxylata*. In addition, VIM enzymes were detected in one *Citrobacter freundii*, one *Leclercia adecarboxylata*, two *Pseudomonas putida* group, one *Pseudomonas stutzeri*, one *Pseudomonas pseudoalcaligenes*, one *Pseudomonas pharmacofabricae*, one *Comamonas aquatica*, one *Comamonas jiangduensis*, one *Pseudomonas mendocina* and one *Pseudomonas oleovorans*/*pseudoalcaligenes* (10.68% of wastewater isolates). Carbapenem-hydrolyzing class D β-lactamases were the most common carbapenemases reported in this study. OXA-48 was detected in 42 strains (40.77%), including enterobacteria, *Aeromonas hydrophila* and *Shewanella putrefaciens*. Furthermore, OXA-181 was detected in three strains, including two *Klebsiella pneumoniae* and one *Escherichia coli*. OXA-23 was detected in only one *Acinetobacter baumannii* isolate. The co-production of two carbapenemases was also reported. Four *Shewanella putrefaciens*, two *Citrobacter braakii*, one *Pseudomonas alcaliphila* and one *Pseudomonas oleovorans* isolate co-harbored *bla*_OXA-48_ and *bla*_VIM_ genes. Furthermore, three *Pseudomonas aeruginosa* isolates presented mutations in their *oprD* genes, leading to imipenem resistance.

**Figure 3:**
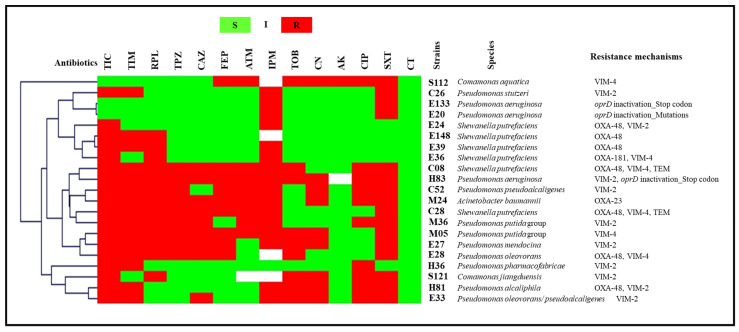
Antibiogram results and resistance mechanisms of glucose-non-fermenting GNB wastewater isolates clustered using the MultiExperimentViewer (MEV) software version 4_6_2. AK, amikacin; ATM, aztreonam; CAZ, ceftazidime; CIP, ciprofloxacin; CN, gentamicin; CT, colistin; FEP, cefepime; I, intermediate; IPM, imipenem; PRL, piperacillin; R, resistant; S, susceptible; SXT, trimethoprim/ sulfamethoxazole; TIC, ticarcillin; TIM, ticarcillin/clavulanate; TOB, tobramycin; TPZ, piperacillin/tazobactam; Strains’ codes: C, cancer hospital; E, environment; H, university hospital; M, maternity hospital; S, public hospital.

**Figure 4:**
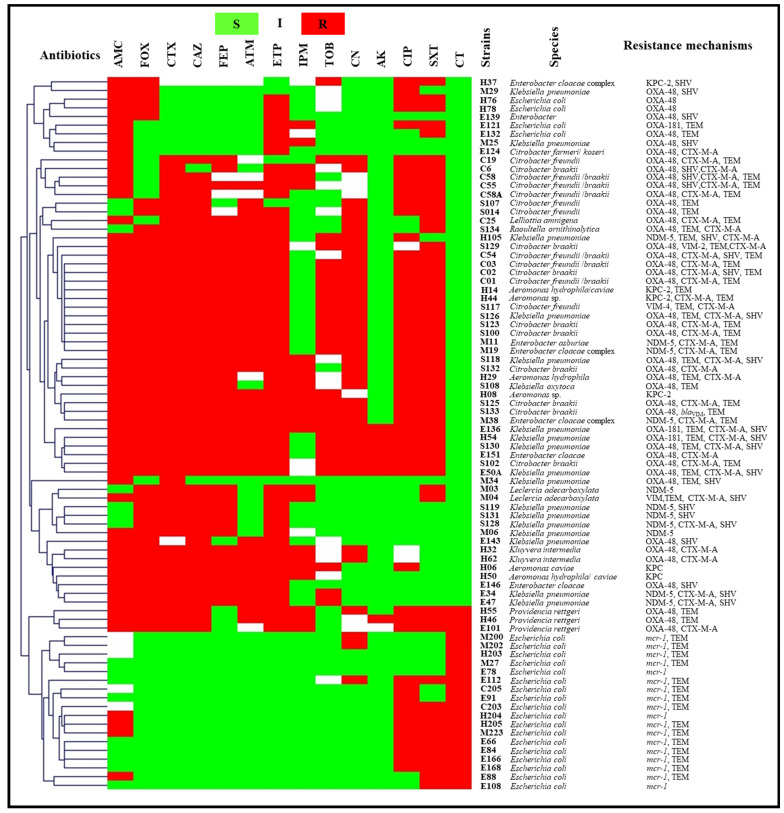
Antibiogram results and resistance mechanisms of enterobacteria and aeromonads isolates from wastewater clustered using the MultiExperimentViewer (MEV) software version 4_6_2. AK, amikacin; AMC, amoxicillin/clavulanate; ATM, aztreonam; CAZ, ceftazidime; CIP, ciprofloxacin; CN, gentamicin; CTX, cefotaxime; CT, colistin; ETP, ertapenem; FEP, cefepime; FOX, cefoxitin; I, intermediate; IPM, imipenem; PRL, piperacillin; R, resistant; S, susceptible; SXT, trimethoprim/ sulfamethoxazole; TIC, ticarcillin; TIM, ticarcillin/clavulanate; TOB, tobramycin; TPZ, piperacillin/tazobactam; Strains’ codes: C, cancer hospital; E, environment; H, university hospital; M, maternity hospital; S, public hospital.

Acquired colistin resistance was observed in 18 *Escherichia coli* isolates, and PCR results revealed that they were all positive for the *mcr-1* gene. It should be noted that most enterobacterial isolates co-expressed TEM, SHV and/or CTX-M-A β-lactamases. Identification, antibiotics susceptibility profiles and colistin resistance mechanisms of wastewater isolates are presented in Figure 3 and Figure 4.

### 2.4. Multilocus Sequence Typing

**Figure 5:**
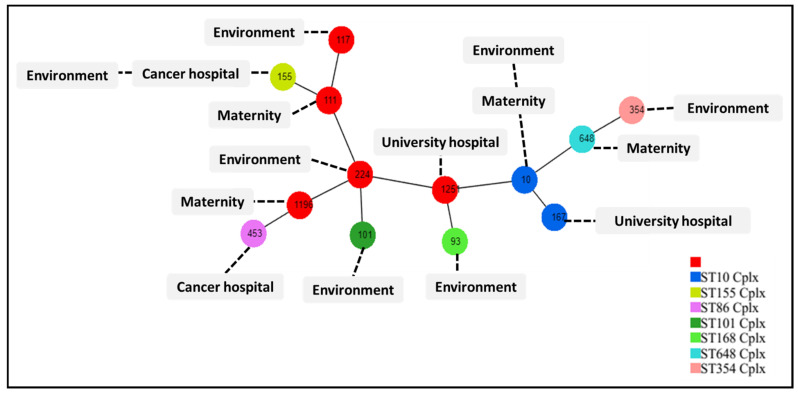
MLST dataset generated using PHYLOViZ Online, indicating STs of our *mcr-1* positive *E. coli* isolates. The numbers in the circles indicate the Sequence Types (ST).

MLST analysis revealed that our *mcr-1* positive *Escherichia coli* isolates belonged to 13 sequence types, namely the high-risk international clones ST10 and ST101, ST93, ST111, ST117, ST155, ST167, ST224, ST354, ST453, ST648, ST1196 and ST1251 (Figure 5).

## 3. Discussion

Antimicrobial resistance has emerged as a major public health concern, not only by health organizations but also by major regulatory, economic and political bodies [13]. This phenomenon is exemplified by the rapid emergence and spread of carbapenem- and colistin-resistance among GNBs. Carbapenems and colistin are undoubtedly among the few available medicines used as a last resort for the treatment of multidrug-resistant (MDR) GNB infections [8,9], and resistance to these compounds is therefore of crucial importance. Indeed, antibiotic resistance is no longer considered as exclusively a clinical settings’ problem. Currently, it is addressed by two complementary concepts, the “One Health” and “Global Health” approaches. The first focuses on local transmission among connected habitats, whereas the second focuses on the broader (even worldwide) transmission of resistant organisms and resistance genes [13]. Within the latter, water environments may play a critical role. In the present study, we aimed to confirm the role of aquatic environments as reservoirs of last resort antibiotic-resistant GNBs.

We previously reviewed the worldwide epidemiology of carbapenemases and *mcr* genes in aqueous ecosystems and reported their broad dissemination among different water types around the world [7,14]. In this study, we investigated the occurrence of carbapenem- and colistin-resistant GNBs in water from different origins. Surveillance of MDR bacteria in the surrounding environment could have a significant impact on the well-being of humans, animals and plants. The main objectives were to assess the risk of MDR bacteria transmission via environmental routes, estimating the evolution of antibiotic resistance, and evaluating the level of antibiotic resistance in the population [15].

In this study we targeted wastewater and tap water, which respond to the aforementioned goals. The first provides an idea regarding the levels of resistance entering the environment, and the second represents a relevant exposure pathway that could help to assess the risk of transmission [15]. Our findings are very significant, as we detected a high level of antibiotic resistance in both wastewater and hospital tap water.

Regarding carbapenem-resistance, here we report the first detection of OXA-48 and VIM-2 enzymes in tap water bacteria and the first NDM-5 and KPC-2-producing isolates from wastewater in Algeria. OXA-48 carbapenemase production was the most common mechanism of carbapenem resistance detected in this study. It has been suggested that this enzyme has been endemic in Algeria since 2012, although there was no data about it at that time [16]. Later, OXA-48 producers were detected in several Algerian cities [17]. In Batna, the region where our study was conducted, this carbapenemase has been detected in different *Enterobacterales* isolates from several origins, including a hospital outbreak [18], in a hospital environment [19,20], in a community-acquired infection [21], in white storks [22,23], coins [24], pigeons [25,26] and fresh vegetables [27].

In addition, we previously conducted a study at the public hospital in 2018, in which a level of resistance to carbapenems which is comparable to that reported here was detected [28], suggesting that the presence of such resistant bacteria in hospital wastewater is not a temporary event but rather a persistent problem. Indeed, the present study, along with the aforementioned reports, confirms the widespread dissemination of OXA-48 producers in Batna and reinforces the suggestion by Poirel and colleagues regarding the endemicity of this enzyme in Algeria. However, additional investigations in other Algerian cities are required to be able to assess the extent of the dissemination of this enzyme.

Other carbapenemases were also detected in this study, both in tap water and wastewater, namely the KPC, VIM and NDM variants. Little data are available concerning the detection of these enzymes in Algeria in comparison with OXA-48 [17]. In the province of Batna, with the exception of VIM-2 and VIM-4, which were detected in hospital cockroaches and human infections, respectively [19,29], no other class A or B carbapenemase has been reported. Thus, the presence of such carbapenemases in hospital and environmental wastewater could originate from their carriage in human and animal gut microbiota. Regarding colistin resistance, *mcr-1* was detected only in *E. coli* isolates obtained from wastewater collected from three hospitals and from the environment. However, colistin-resistant enterobacteria isolated from tap water was potentially related to mutational mechanisms. In addition, we reported the first *mcr-5* positive *Cupriavidus gilardii* through its isolation from well water [12].

The *mcr-1* positive *E. coli* isolates belonged to different sequence types (Figure 5), revealing a heterogenous spread of this resistance gene in this species and suggesting that clonal expansion is not involved in the spread of *mcr-1* in this region. This was previously demonstrated by Shen and colleagues [30]. In addition, MLST analysis suggests that these strains originate from human carriage, as most of our strains belonged to phylogenetic groups A and B1, which are known to represent human commensal *E. coli* [31], and especially given that we have no data regarding the detection of *mcr-1* carrying *E. coli* in human or animal infections in Batna.

Finally, comparing phenotypic and molecular results showed discordance in several isolates. In particular, this was the case in those susceptible to carbapenems with confirmed carbapenemase production and colistin resistant *K. pneumoniae* with no detected mechanism. Indeed, this could be due to the expression problems along with the presence of non-investigated or unknown colistin resistance mechanisms [32]. Indeed, such discordance has already been reported among carbapenemase producers in several previous studies including KPC-producers [33], MBL-producers [34] and OXA-48-producers [35]. The detection of those so-called “hidden” carbapenemases is of critical importance considering their participation in the silent dissemination of resistance genes either in hospitals or in the community.

Regardless of antibiotic resistance, the high bacterial load in tap water from the maternity hospital and the cancer hospital represents a major concern and could have a negative effect on patients’ health. This could be attributed to the direct use of well water without any treatment before its distribution. An antibacterial treatment such as chlorination is highly recommended to reduce bacterial loads, as is the case in the university hospital and the public hospital where no isolates were detected in most samples.

Water is the most crucial natural resource on Earth. However, it is continuously polluted with different types of contaminants [36], including antibiotic resistance genes and resistant bacteria [37]. The presence of various anthropogenic pollutants, especially in aqueous environments, is also potentially hazardous and makes understanding the emergence, development and spread of antibiotic-resistant bacteria highly challenging [38]. At the same time, water contamination with antibiotic-resistant bacteria could have serious implications for all organisms, as well as for the environment, as it is used in a range of ways, including for recreational purposes, drinking, and crop irrigation. Understanding the sources and mechanisms of the emergence and spread of antibiotic-resistant bacteria, especially those resistant to last resort molecules, in the aquatic environment could contribute to the development of new strategies for the surveillance and reduction of these harmful organisms in water systems [39].

The release of antibiotic-resistant bacteria into groundwater may occur in several ways, including runoff, surface water, sewage pipes and soil, especially after the application of contaminated fertilizers [7]. In addition, several factors could affect the transmission of resistant bacteria and resistance genes into groundwater such as pH, temperature, salinity, redox potential, organic matter, soil properties, the depth of the water table, and the physiological properties of the bacteria [39]. The wells sampled in this study are located in urban areas away from agricultural regions; however, the presence of contamination points should not be excluded. In tap water distribution systems, other factors can promote the presence of antibiotic resistance including pipe biofilm, loose deposits, and suspended solids [39].

In spite of the detection of high levels of resistance in well water and tap water, wastewater remains the largest reservoir of resistance to last-resort antibiotics. Unfortunately, hospital wastewater is directly discharged without any treatment into surface water bodies, presenting a serious public health concern. This ends up in rural areas where it may be used for irrigation. In addition, birds, including migratory species, use this water for feeding. Thus, the resistant bacteria and genes which are detected can reach these birds’ intestinal tracts, resulting in another resistance reservoir and route of dissemination. Consequently, the regulation of water contamination and the development of effective treatment strategies for eliminating antibiotic resistance are required.

## 4. Materials and Methods

### 4.1. Sampling

Between January 2018 and October 2019, 207 water samples were collected from different sources in the province of Batna in Algeria. In the present study, we included hospital sewage, wastewater discharged into the environment, water from wells supplying hospitals, water tanks, and hospital tap water. Sampling sites and dates are presented in Table S1. Regarding wastewater, samples were collected from the four major hospitals in the city of Batna, including the public hospital, the university hospital, a maternity hospital, and the cancer hospital. In addition, discharged wastewater samples were collected throughout Wadi El Gourzi, which receives wastewater released from different parts of the city of Batna. Samples were collected in one-liter sterile glass bottles.

The tap water in the hospitals that were included in this study is supplied from wells. One-liter samples were collected from wells, water tanks and taps in different wards and rooms. Taps were first disinfected by bleach, then sterilized using a flame. The water was then allowed to flow until the tap cooled. Subsequently, one liter of water was collected in a sterile glass bottle. The chlorinated tap water samples were collected in sterile glass bottles containing sodium thiosulfate at a final concentration of 17.5 mg/L. All samples were kept at 4°C until processing, which took place within four hours.

### 4.2. Samples’ Processing and Bacterial Isolation

#### 4.2.1. Wastewater

After manual homogenization, 200 mL of each sample was centrifugated at 7000 rpm (7943× *g*) for 15 min at 4 °C. Pellets were then subjected to selective isolation of carbapenem- and colistin-resistant GNBs. First, one inoculation loop (10 µL) of each pellet was streaked on MacConkey agar plates supplemented with the following four different antibiotic combinations: 64 µg/mL of vancomycin and 4 µg/mL of imipenem, 64 µg/mL of vancomycin and 2 µg/mL of ertapenem, 10 µg/mL of daptomycin and 2 µg/mL of colistin, 10 µg/mL of daptomycin and 2 µg/mL of colistin, and 2 µg/mL of ertapenem. At the same time, 2 mL from each pellet was enriched in brain heart infusion broth with the aforementioned antibiotic combinations, and sterile paraffin oil was added to the tubes containing vancomycin and ertapenem in order to create anaerobic conditions. After overnight incubation, one inoculation loop of each positive tube was inoculated onto MacConkey agar plates containing the same selective antibiotics. Plates were aerobically incubated for 18 to 24 h at 37 °C. Morphologically distinguishable colonies from all plates were purified and conserved until subsequent processing.

#### 4.2.2. Well Water and Tap Water

One-liter water samples were filtered through sterile cellulose membranes (0.45 µm pore size), and then filters were placed on MacConkey agar plates and aerobically incubated overnight at 37 °C. Subsequently, representative colonies were purified on the same medium. Afterwards, each filter was sub-cultured using the replica plating method on MacConkey agar plates supplemented by the aforementioned combinations of antibiotics, and representative colonies were purified and conserved until subsequent processing.

### 4.3. Bacterial Identification

All isolates were identified using matrix-assisted laser desorption and ionization time-of-flight mass spectrometry (MALDI-TOF MS), as previously described [40]. Briefly, colonies from pure culture grown on sheep blood agar were picked and spotted onto the steel target plate on thin films in four replicates. Subsequently, each spot was layered with 1.5 µL of saturated α-cyno-hydroxy-cinnamic acid in 50% acetonitrile, and 2.5% trifluoracetic-acid and allowed to air dry at room temperature before analysis. Which was processed on a Microflex LTII (Bruker Daltonics, Bremen, Germany). The results of the mass spectrum were imported into MALDI Biotyper 3.0 software (Bruker Daltonics) and analyzed against the spectra of bacteria included in the Bruker database and regularly updated MEPHI databases. Non-inoculated matrix solution and *Escherichia coli* DH5alpha (ref 255343, Bruker Daltonics) were used as negative and positive controls, respectively.

In addition, isolates that could not be identified by MALDI-TOF MS were subjected to 16S rRNA encoding gene sequencing, as previously described [41]. Sequences were analyzed using the CodonCode Aligner Software version 9.0.1, then blasted against the constantly updated NCBI Reference 16S rRNA sequences database refseq_rna (https://blast.ncbi.nlm.nih.gov/Blast.cgi?PROGRAM=blastn&PAGE_TYPE=BlastSearch&LINK_LOC=blasthome (accessed on 10 July 2022)).

### 4.4. Antibiotic Susceptibility Testing and Phenotypic Detection of β-Lactamase Production

All of the obtained strains were examined for their antibiotic sensitivity by using the disc diffusion method as described by the Antibiogram Committee of the French Microbiology Society [42]. The following antibiotics were tested: ticarcillin (75 µg), piperacillin (75 µg), ticarcillin-clavulanate (75/10 µg), amoxicillin-clavulanate (20/10 µg), piperacillin-tazobactam (75/10 µg), cefoxitin (30 µg), cefalotin (30 µg), cefotaxime (30 µg), ceftazidime (30 µg), cefepime (30 µg), aztreonam (30 µg), ertapenem (10 µg), imipenem (10 µg), tobramycin (10 µg), gentamicin (15 µg), amikacin (30 µg), ciprofloxacin (5 µg), cotrimoxazole (1.25/23.75 µg), and colistin (50 µg). Inhibition zone diameters were interpreted according to the European Committee on Antimicrobial Susceptibility Testing (EUCAST).

Strains were tested for any production of carbapenemases and extended-spectrum β-lactamases (ESBLs) using the modified CarbaNP-test (MCNP-test) and the double-disc synergy test, respectively [43,44].

In addition, minimum inhibitory concentrations (MICs) of ertapenem and imipenem were determined using the E-test. Colistin MICs were determined using broth microdilution for strains showing a reduced inhibition zone diameter.

### 4.5. Molecular Characterization of β-Lactam- and Colistin Resistance Mechanisms

Genomic DNA extraction was conducted using the EZ1 biorobot (Qiagen, Hilden, Germany), using the EZ1 DNA tissue kit (Qiagen) and the bacterial protocol card. β-lactam-resistant strains were screened for the occurrence of the most common β-lactam-resistance encoding genes detected in Algeria including *bla*_KPC_, *bla*_NDM_, *bla*_VIM_, *bla*_OXA-23_, *bla*_OXA-24_, *bla*_OXA-48_, *bla*_OXA-58_, *bla*_CTX-M_, *bla*_TEM_ and *bla*_SHV_ genes by real time PCR, as previously described [28].

Colistin-resistant wastewater isolates and all tap water isolates were tested for the presence of *mcr-1*, *mcr-2*, *mcr-3*, *mcr-4*, *mcr-5* and *mcr-8* genes by real time PCR, as previously described [45,46] (File S2). Positive strains for carbapenemases or *mcr* encoding genes were confirmed by standard PCR. Amplified products were sequenced using the Sanger sequencing method and Big Dye terminator chemistry on an ABI 3500XL automated sequencer (Applied biosystems, Foster City, CA, USA). Sequences were analysed using the CodonCode Aligner Software and then blasted against the ARG-ANNOT (Antibiotic Resistance Genes-ANNOTation) database [47]. In addition, imipenem-resistant *Pseudomonas aeruginosa* isolates were subjected to the amplification of the *oprD* gene using previously described primers [48]. The fully amplified and sequenced genes were compared by alignment with the reference sequence of the *P. aeruginosa* PAO1 strain (GenBank accession no. NC_002516.2).

Furthermore, mutational mechanisms of colistin resistance in colistin-resistant *Klebsiella pneumoniae* and *Enterobacter cloacae* isolates were investigated by PCR and sequencing using the primers described in File S2. The fully amplified and sequenced genes were compared with the reference sequences of *Klebsiella pneumoniae* subsp. *pneumoniae* MGH 78578 (GCF_000016305) and *Enterobacter cloacae* subsp. *cloacae* ATCC 13047 (GCF_000025565), respectively.

### 4.6. Multilocus Sequence Typing

Multilocus sequence typing of the *mcr-1* positive *Escherichia coli* isolates was performed using the Warwick scheme by PCR amplification and sequencing of the internal fragments of the seven housekeeping genes, *adk*, *fumC*, *gyrB*, *icd*, *mdh*, *purA* and *recA*. Alleles and sequence types were determined according to the MLST database (https://enterobase.warwick.ac.uk/species/index/ecoli (accessed on 10 July 2022)).

## 5. Conclusions

Water is a medium which connects different ecological systems and can easily transmit resistant bacteria and promote horizontal gene transfer. In this study, we observed that aquatic environments are reservoirs of last-resort antibiotic-resistant GNBs, allowing their possible widespread dissemination into the environment. These aquatic environments therefore currently present a major problem which threatens public health, animal health, the environment, and food security.

The interdependence between the environment and the health of humans, animals and plants makes the monitoring and control of the antibiotic resistance phenomenon indispensable. Hence the urgent need for interdisciplinary collaboration to establish effective strategies to control and prevent the spread of such bacteria. In addition, it seems clear that wastewater is an important reservoir of resistant bacteria and should, therefore, be a primary target for control and prevention efforts. The development of efficient wastewater treatment methods to eliminate or at least decrease antibiotic-resistant bacteria and antibiotic-resistance genes in wastewater is strongly recommended.

## Data Availability

Not applicable.

## Supplementary Materials

The following supporting information can be downloaded at: https://www.mdpi.com/article/10.3390/antibiotics11101314/s1, Figure S1: Identification results. CH, cancer hospital; ENV, environment; MH, maternity hospital; PH, public hospital; UH, university hospital; Figure S2: Antibiogram results. AK, amikacin; AMC, amoxicillin/ clavulanate; ATM, aztreonam; CAZ, ceftazidime; CIP, ciprofloxacin; CN, gentamicin; CTX, cefotaxime; CT, colistin; ETP, ertapenem; FEP, cefepime; I, intermediate; IPM, imipenem; KF, cefalotin; R, resistant; S, susceptible; SXT, cotrimoxazole; TOB, tobramycin; Table S1. Sample sources, numbers and sampling dates; File S1: Identification, susceptibility testing, resistance mechanisms and MLST results obtained in this study; File S2: Primers used for the detection of colistin resistance in this study.
