# Supplementary material for: Dissemination of Carbapenemases and MCR-1 Producing Gram-Negative Bacteria in Aquatic Environments in Batna, Algeria
Source: Antibiotics (Basel). 2022 Sep 27;11(10):1314. doi: 10.3390/antibiotics11101314 (PMC9598638; doi:10.3390/antibiotics11101314)
Supplement: Supplementary file 1 [file antibiotics-11-01314-s001.zip › Supplementary data_Figure S1.pptx]

## Slide 1
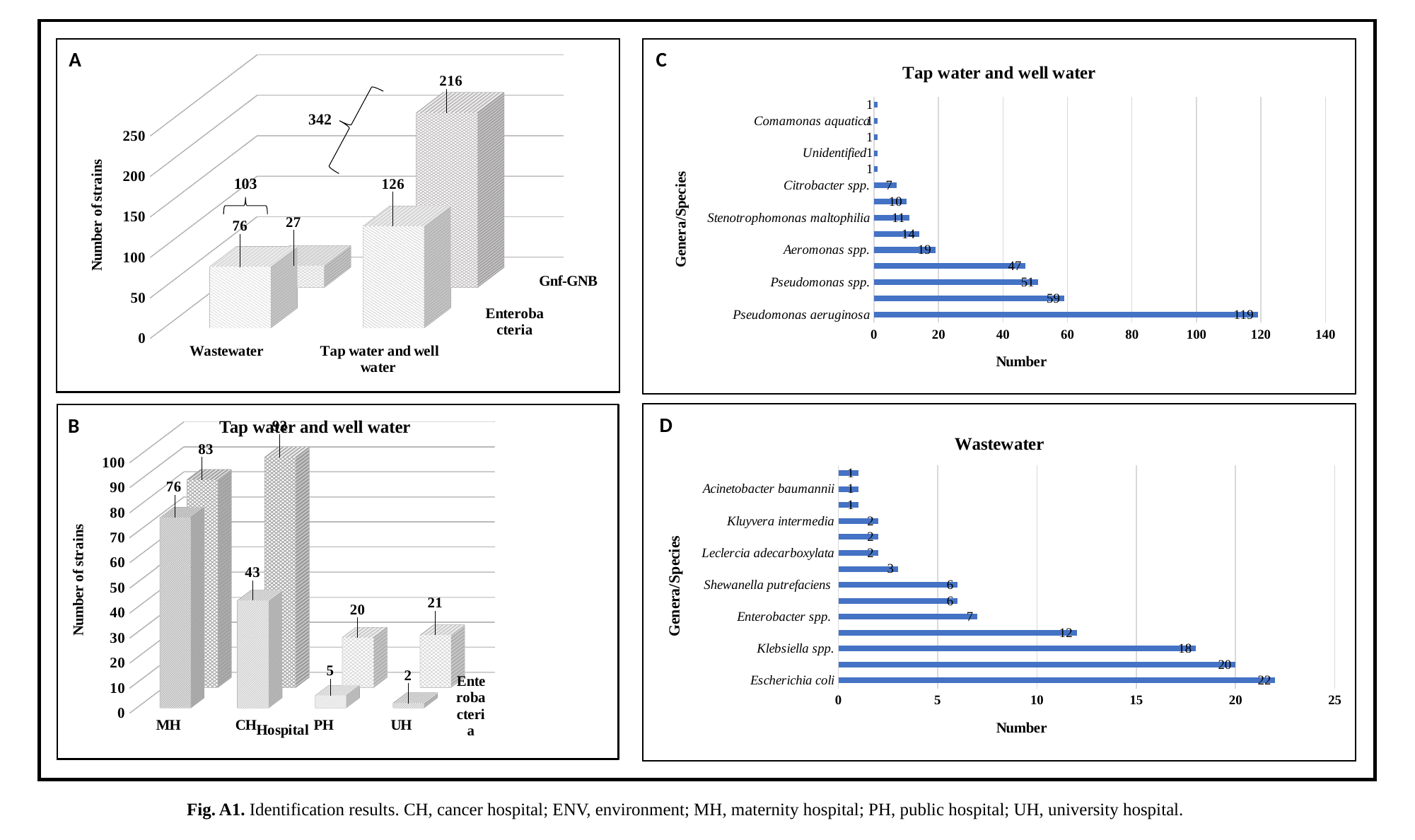

[unsupported chart]
342
103
A
### Chart: Tap water and well water
| Category | |
|---|---|
| Pseudomonas aeruginosa | 119.0 |
| Enterobacter spp. | 59.0 |
| Pseudomonas spp. | 51.0 |
| Klebsiella spp. | 47.0 |
| Aeromonas spp. | 19.0 |
| Acinetobacter spp. | 14.0 |
| Stenotrophomonas maltophilia | 11.0 |
| Escherichia coli | 10.0 |
| Citrobacter spp. | 7.0 |
| Leclercia adecarboxylata | 1.0 |
| Unidentified | 1.0 |
| Cronobacter sp. | 1.0 |
| Comamonas aquatica | 1.0 |
| Cupriavidus gilardii | 1.0 |C
### Chart: Wastewater
| Category | |
|---|---|
| Escherichia coli | 22.0 |
| Citrobacter spp. | 20.0 |
| Klebsiella spp. | 18.0 |
| Pseudomonas spp. | 12.0 |
| Enterobacter spp. | 7.0 |
| Aeromonas spp. | 6.0 |
| Shewanella putrefaciens | 6.0 |
| Providencia rettgeri | 3.0 |
| Leclercia adecarboxylata | 2.0 |
| Comamonas spp. | 2.0 |
| Kluyvera intermedia | 2.0 |
| Raoultella ornithinolytica | 1.0 |
| Acinetobacter baumannii | 1.0 |
| Lelliottia amnigena | 1.0 |
[unsupported chart]
D
B
Tap water and well water
Fig. A1. Identification results. CH, cancer hospital; ENV, environment; MH, maternity hospital; PH, public hospital; UH, university hospital.
