# Supplementary material for: Dissemination of Carbapenemases and MCR-1 Producing Gram-Negative Bacteria in Aquatic Environments in Batna, Algeria
Source: Antibiotics (Basel). 2022 Sep 27;11(10):1314. doi: 10.3390/antibiotics11101314 (PMC9598638; doi:10.3390/antibiotics11101314)
Supplement: Supplementary file 1 [file antibiotics-11-01314-s001.zip › Supplementary data_Figure S2.pptx]

## Slide 1
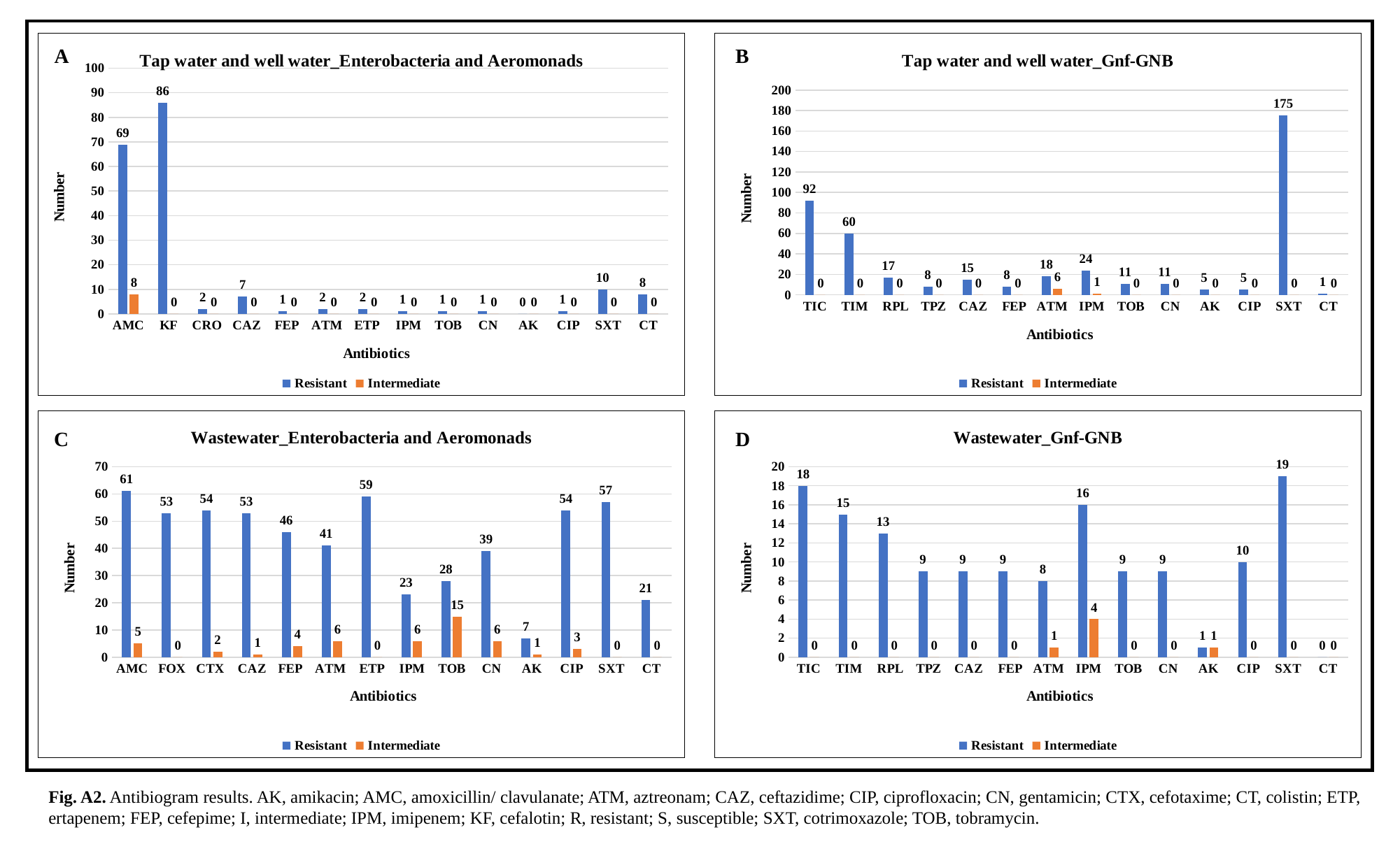

### Chart: Tap water and well water_Gnf-GNB
| Category | Resistant | Intermediate |
|---|---|---|
| TIC | 92.0 | 0.0 |
| TIM | 60.0 | 0.0 |
| RPL | 17.0 | 0.0 |
| TPZ | 8.0 | 0.0 |
| CAZ | 15.0 | 0.0 |
| FEP | 8.0 | 0.0 |
| ATM | 18.0 | 6.0 |
| IPM | 24.0 | 1.0 |
| TOB | 11.0 | 0.0 |
| CN | 11.0 | 0.0 |
| AK | 5.0 | 0.0 |
| CIP | 5.0 | 0.0 |
| SXT | 175.0 | 0.0 |
| CT | 1.0 | 0.0 |
### Chart: Tap water and well water_Enterobacteria and Aeromonads
| Category | Resistant | Intermediate |
|---|---|---|
| AMC | 69.0 | 8.0 |
| KF | 86.0 | 0.0 |
| CRO | 2.0 | 0.0 |
| CAZ | 7.0 | 0.0 |
| FEP | 1.0 | 0.0 |
| ATM | 2.0 | 0.0 |
| ETP | 2.0 | 0.0 |
| IPM | 1.0 | 0.0 |
| TOB | 1.0 | 0.0 |
| CN | 1.0 | 0.0 |
| AK | 0.0 | 0.0 |
| CIP | 1.0 | 0.0 |
| SXT | 10.0 | 0.0 |
| CT | 8.0 | 0.0 |B
A
### Chart: Wastewater_Enterobacteria and Aeromonads
| Category | Resistant | Intermediate |
|---|---|---|
| AMC | 61.0 | 5.0 |
| FOX | 53.0 | 0.0 |
| CTX | 54.0 | 2.0 |
| CAZ | 53.0 | 1.0 |
| FEP | 46.0 | 4.0 |
| ATM | 41.0 | 6.0 |
| ETP | 59.0 | 0.0 |
| IPM | 23.0 | 6.0 |
| TOB | 28.0 | 15.0 |
| CN | 39.0 | 6.0 |
| AK | 7.0 | 1.0 |
| CIP | 54.0 | 3.0 |
| SXT | 57.0 | 0.0 |
| CT | 21.0 | 0.0 |
### Chart: Wastewater_Gnf-GNB
| Category | Resistant | Intermediate |
|---|---|---|
| TIC | 18.0 | 0.0 |
| TIM | 15.0 | 0.0 |
| RPL | 13.0 | 0.0 |
| TPZ | 9.0 | 0.0 |
| CAZ | 9.0 | 0.0 |
| FEP | 9.0 | 0.0 |
| ATM | 8.0 | 1.0 |
| IPM | 16.0 | 4.0 |
| TOB | 9.0 | 0.0 |
| CN | 9.0 | 0.0 |
| AK | 1.0 | 1.0 |
| CIP | 10.0 | 0.0 |
| SXT | 19.0 | 0.0 |
| CT | 0.0 | 0.0 |D
C
Fig. A2. Antibiogram results. AK, amikacin; AMC, amoxicillin/ clavulanate; ATM, aztreonam; CAZ, ceftazidime; CIP, ciprofloxacin; CN, gentamicin; CTX, cefotaxime; CT, colistin; ETP, ertapenem; FEP, cefepime; I, intermediate; IPM, imipenem; KF, cefalotin; R, resistant; S, susceptible; SXT, cotrimoxazole; TOB, tobramycin.
