# Supplementary material for: Dissemination of Carbapenemases and MCR-1 Producing Gram-Negative Bacteria in Aquatic Environments in Batna, Algeria
Source: Antibiotics (Basel). 2022 Sep 27;11(10):1314. doi: 10.3390/antibiotics11101314 (PMC9598638; doi:10.3390/antibiotics11101314)
Supplement: Supplementary file 1 [file antibiotics-11-01314-s001.zip › Supplementary data_File S2.pdf]

File A1. Primers used for the detection of colistin resistance mechanisms in this study.

A. Mutational mechanisms

1. *Klebsiella pneumoniae*

Target gene	PCR Type	Sequences	Program	Reference
PmrA	Standard	pmrA_F ATCTGTTCTCCACCCTGCTC	Annealing T° : 55°C<br>Extension : 1 min 30 s	(Olaitan et al., 2014)
		pmrA_R CATGCTCCACATGTTTCTGG		
PmrB	Standard	pmrB-KP-F1 CGACATTTACAGCTGGGACA		
		PmrB-KP-R2 TTGCGGCCTTCTTAATGTTT		
	Sequencing	pmrB-KP-F1 CGACATTTACAGCTGGGACA	-	
		pmrB-KP-R1 TTTACCACGTCATCCAGCAA		
		pmrB-KP-F2 GAGCTGCTGGCGAAAGTG		
		pmrB-KP-R2 TTGCGGCCTTCTTAATGTTT		
PhoP	Standard	phoP_F GAAGCGAACCCGTAATGACA	Annealing T° : 55°C<br>Extension : 1 min 30 s	
		phoP_R AACAGCCGTTTATATTTGCGTA		
PhoQ	Standard	phoQ_F GCAGGTGTCTGACAGGGATT	Annealing T° : 55°C<br>Extension : 1 min 30 s	
		phoQ_R AGGCTGAATACCCACAGGAC		
	Sequencing	phoQ_F GCAGGTGTCTGACAGGGATT	-	
		phoQ_R AGGCTGAATACCCACAGGAC		
		phoQ_F1 ATTGACCCCTTTGCGCTGATA		
		phoQ_R1 ATCCGCGAACAGGGCGACGACT		
mgrB	Standard and sequencing	mgrB_F1 ATTCTGCCGCTTTTGCTG	Annealing T° : 51°C<br>Extension : 74°C/90 s	
		mgrB_R1 CGTTTTGAAACAAGTCGATGA		

2. *Enterobacter cloacae*

Target gene	PCR Type	Sequences	Program	Reference
PmrA	Standard and sequencing	pmrA_F ATGAAACTACTGATAGTTGAAGA<br>pmrA_R CATTTMTGDCTMTCCAGMCG	Annealing T° : 55°C<br>Extension : 1 min 30 s	Our lab
PmrB	Standard and sequencing	pmrB_F ATGAACAGYATGCGYCGGCG<br>pmrB_R CTCAGRCCMAGSCCRCTR		
PhoP	Standard and sequencing	phoP_F ATGCGCGTACTGGTTGTTGA<br>phoP_R GCGTAWTTCRAACAGRTARC		
PhoQ	Standard and sequencing	phoQ_F CCCTYTCRCTGCGGGTTCG<br>phoQ_R TCGTACTGRTCGACRATYTC		
mgrB	Standard and sequencing	mgrB_F CGGTTTACTCTATGAAACAAGTGC<br>mgrB_R GCGAAGGAAGGAAATCACCT		

B. *mcr* genes

Target gene	PCR Type	Sequences	Reference
mcr-1	RT PCR	F: GCAGCATACTTCTGTGTGGTAC	(Chabou et al., 2016)
		R: ACAAAGCCGAGATTGTCCGCG	
		Probe : FAM-GACCGCGACCGCCAATCTTACC-TAMRA	
	Standard and sequencing	F: GCAGCATACTTCTGTGTGGTAC	(Touati et al., 2019)
		R: TATGCACGCGAAAGAAACTGGC	
mcr-2	RT PCR	F: CTGTGCCGTGTATGTTCAGC	
		R: TTATCCATCACGCCTTTTGAG	
		Probe: VIC-TGACCGCTTGGGTGTGGGTA-TAMRA	
mcr-3	RT PCR	F: TGAATCACTGGGAGCATTAGGGC	
		R: TGCTGCAAACACGCCATATCAAC	
		Probe: FAM-TGCACCGGATGATCAGACCCGT-TAMRA	
mcr-4	RT PCR	F: GCCAACCAATGCTCATACCCAAAA	
		R: CCGCCCCATTCGTGAAAACATAC	
		Probe : FAM-GCCACGGCGGTGTCTCTACCC-TAMRA	
mcr-5	RT PCR	F: TATCCCGCAAGCTACCGACGC	
		R: ACGGGCAAGCACATGATCGGT	
		Probe: FAM-TGCGACACCACCGATCTGGCCA-TAMRA	
	Standard and sequencing	F: ATGCGGTTGTCTGCATTTATC	(Borowiak et al., 2017)
		R: TCATTGTGGTTGTCCTTTTCTG	
mcr-8	RT PCR	F: TCCGGGATGCGTGACGTTGC	(Nabti et al., 2020)
		R: TGCTGCGCGAAT-GAAGACG	
		Probe: FAM-TCATGGAGAATCGCTGGGG-GAAAGC-TAMRA	

References

Borowiak, M.; Fischer, J.; Hammerl, J.A.; Hendriksen, R.S.; Szabo, I. and Malorny, B., 2017. Identification of a novel transposon-associated phosphoethanolamine transferase gene, *mcr-5*, conferring colistin resistance in d-tartrate fermenting *Salmonella enterica* subsp. *enterica* serovar Paratyphi B. Journal of Antimicrobial Chemotherapy 72(12), 3317-3324.

Chabou, S.; Leangapichart, T.; Okdah, L.; Le Page, S.; Hadjadj, L. and Rolain, J.M., 2016. Real-time quantitative PCR assay with Taqman® probe for rapid detection of MCR-1 plasmid-mediated colistin resistance. New Microbes New Infect 13, 71-74.

Nabti, L.Z.; Sahli, F.; Ngaiganam, E.P.; Radji, N.; Mezaghcha, W.; Lupande-Mwenebitu, D.; Baron, S.A.; Rolain, J.-M. and Diene, S.M., 2020. Development of real-time PCR assay allowed describing the first clinical *Klebsiella pneumoniae* isolate harboring plasmid-mediated colistin resistance *mcr-8* gene in Algeria. J Glob Antimicrob Resist 20, 266-271.

Olaitan, A.O.; Diene, S.M.; Kempf, M.; Berrazeg, M.; Bakour, S.; Gupta, S.K.; Thongmalayvong, B.; Akkhavong, K.; Somphavong, S.; Paboriboune, P.; Chaisiri, K.; Komalamisra, C.; Adelowo, O.O.; Fagade, O.E.; Banjo, O.A.; Oke, A.J.; Adler, A.; Assous, M.V.; Morand, S.; Raoult, D. and Rolain, J.M., 2014. Worldwide emergence of colistin resistance in *Klebsiella pneumoniae* from healthy humans and patients in Lao PDR, Thailand, Israel, Nigeria and France owing to inactivation of the PhoP/PhoQ regulator *mgrB*: an epidemiological and molecular study. Int J Antimicrob Agents 44(6), 500-507.

Touati, M.; Hadjadj, L.; Berrazeg, M.; Baron, S. and Rolain, J.M., 2019. Emergence of *Escherichia coli* harboring *mcr-1* and *mcr-3* gene in North West Algerian farmlands. J Glob Antimicrob Resist.
