# Supplementary material for: Dissemination of Carbapenemases and MCR-1 Producing Gram-Negative Bacteria in Aquatic Environments in Batna, Algeria
Source: Antibiotics (Basel). 2022 Sep 27;11(10):1314. doi: 10.3390/antibiotics11101314 (PMC9598638; doi:10.3390/antibiotics11101314)
Supplement: Supplementary file 1 [file antibiotics-11-01314-s001.zip › Supplementary data_Table S1.pdf]

**Table S1.** Sample sources, numbers and sampling dates.

| Sampling date | Source | Type | Number |
|---|---|---|---|
| **20/01/2019** | University hospital | Wastewater | 08 |
| **21/02/2019** | Cancer hospital | | 05 |
| **19/05/2019** | Wadi El Gourzi | | 13 |
| **08/09/2019** | Public hospital | | 03 |
| **01/10/2019** | Maternity hospital | | 05 |
| **17/10/2019** | University hospital | | 01 |
| | **Total** | | **35** |
| **03/09/2019** | Public hospital | Tap water and well water | 27 |
| **08/09/2019** | Public hospital | | 07 |
| **16/09/2019** | Maternity hospital | | 30 |
| **22/09/2019** | Maternity hospital | | 03 |
| **23/09/2019** | Maternity hospital | | 05 |
| **20/09/2019** | University hospital | | 20 |
| **03/10/2019** | University hospital | | 20 |
| **07/10/2019** | Cancer hospital | | 23 |
| **14/10/2019** | University hospital | | 21 |
| **15/10/2019** | University hospital | | 06 |
| **16/10/2019** | Cancer hospital | | 10 |
| | **Total** | | **172** |
